# Closed Genome Sequence of Escherichia coli K-12 Group Strain C600

**DOI:** 10.1128/MRA.01052-18

**Published:** 2019-01-10

**Authors:** Anna Allué-Guardia, Emmanuel C. Nyong, Sara S. K. Koenig, Sean M. Vargas, James L. Bono, Mark Eppinger

**Affiliations:** a Department of Biology, University of Texas at San Antonio, San Antonio, Texas, USA; b South Texas Center for Emerging Infectious Diseases (STCEID), San Antonio, Texas, USA; c U.S. Department of Agriculture, Agricultural Research Service (USDA-ARS), Clay Center, Nebraska, USA; University of Delaware

## Abstract

Escherichia coli strain C600 is a prototypical K-12 derived laboratory strain which has been broadly used for molecular microbiology and bacterial physiology studies since its isolation in 1954. Here, we present the closed genome sequence of E. coli strain C600, retrieved from the American Type Culture Collection (ATCC 23724).

## ANNOUNCEMENT

Escherichia coli strain C600 (Migula) Castellani and Chalmers (ATCC 23724) is a bacteriophage λ-sensitive strain first described in 1954 (1) and derived from progenitor strain K-12, which was originally isolated in 1922 (2). Since its isolation in 1954, strain C600 has been used as a model E. coli strain in laboratories worldwide for molecular microbiology, genetic, and physiology studies (3, 4). Its genome can incorporate foreign DNA by transformation and conjugation (5, 6). Strain C600 is also susceptible to phage transduction and serves as a prime bacterial host in bacteriophage infectivity assays, producing stable lysogens (7, 8). Recovered Shiga-toxigenic lysogens in the C600 genome background that were transduced by Shiga toxin (Stx)-converting bacteriophages have shown an increased ability to produce Shiga toxin under spontaneous noninduced conditions (8) and when cocultured with Stx-producing E. coli (9, 10).

E. coli strain C600 was cultured overnight in Luria-Bertani broth (37°C, 180 rpm), and total genomic DNA was extracted using the QIAamp DNA minikit (Qiagen), following the manufacturer's instructions. To generate a high-quality closed genome sequence, we pursued a hybrid approach using long-read PacBio RS II and short-read Illumina MiSeq sequencing. Briefly, for PacBio sequencing, genomic DNA was sheared into 20-kb fragments using a g-Tube device (Covaris), and a library was prepared based on the 20-kb PacBio sample preparation protocol and sequenced using P6/C4 chemistry on a single-molecule real-time (SMRT) cell with a 240-min collection time. The 113,777 long reads (*N*_50_ = 7,800) were assembled *de novo* using the PacBio Hierarchical Genome Assembly Process (HGAP v3.0) (11) with default parameters in SMRT Analysis v2.3.0 and consensus polishing with Quiver (11). For Illumina sequencing, a paired-end 2 × 250-bp library was prepared using the Nextera XT DNA library preparation kit (Illumina) and sequenced using the 500-cycle MiSeq reagent kit v2 (Illumina), yielding a total of 697,116 paired-end reads. Illumina reads were used for sequence error correction of the PacBio assembly, using Pilon v1.18 with default settings (12).

The assembly yielded a circular chromosome of 4,599,824 bp, with a coverage of 107× and a GC content of 50.8%. The chromosomal origin of replication, *oriC*, was designated the start point (+1) of the closed molecule and determined using the Web-based tool Ori-Finder (13). Genome annotation with the NCBI Prokaryotic Genome Annotation Pipeline (PGAP) (14) predicted a total of 4,636 protein-coding sequences, 22 rRNAs, 86 tRNAs, and 15 noncoding RNAs (ncRNAs).

Strain C600 belongs to sequence type 10 (ST10), as inferred from MLST (v2.0) (15) using the Achtman scheme (16). Prophage profiling with PHASTER identified six prophage-associated regions, including three intact prophages and three incomplete phage remnants (17). *In silico* antimicrobial susceptibility testing using ResFinder (v3.0) with default settings detected no antibiotic resistance genes (18).

### Data availability.

The annotated chromosome has been deposited in NCBI GenBank under the accession number CP031214. Illumina and PacBio reads are available under the accession numbers SRX4909245 and SRX4908799, respectively, in the Sequence Read Archive (SRA).
